# Exploring the Feasibility of Use of An Online Dietary Assessment Tool (myfood24) in Women with Gestational Diabetes

**DOI:** 10.3390/nu10091147

**Published:** 2018-08-23

**Authors:** Carla Gianfrancesco, Zoe Darwin, Linda McGowan, Debbie M. Smith, Roz Haddrill, Michelle Carter, Eleanor M. Scott, Nisreen A. Alwan, Michelle A. Morris, Salwa A. Albar, Janet E. Cade

**Affiliations:** 1 Nutritional Epidemiology Group, School of Food Science and Nutrition, University of Leeds, Leeds LS2 9JT, UK; carla.gianfrancesco@sth.nhs.uk (C.G.); michellecarter.uk@hotmail.co.uk (M.C.); 2 Sheffield Diabetes and Endocrine Centre, Sheffield Teaching Hospitals NHSF Trust, Sheffield S5 7AU, UK; 3 School of Healthcare, University of Leeds, Leeds LS2 9JT, UK; Z.J.Darwin@leeds.ac.uk (Z.D.); L.McGowan@leeds.ac.uk (L.M.); 4 School of Social and Health Sciences, Leeds Trinity University, Leeds LS18 5HD, UK; D.Smith@leedstrinity.ac.uk; 5 Department of Nursing, Midwifery & Health, Northumbria University, Newcastle upon Tyne NE7 7XA, UK; Roz.Haddrill@northumbria.ac.uk; 6 Leeds Institute of Cardiovascular and Metabolic Medicine, University of Leeds, Leeds LS2 9JT, UK; e.m.scott@leeds.ac.uk; 7 Academic Unit of Primary Care and Population Sciences, Faculty of Medicine, University of Southampton, Southampton General Hospital, Southampton SO16 6YD, UK; n.a.alwan@soton.ac.uk; 8 NIHR Southampton Biomedical Research Centre, University of Southampton and University Hospital Southampton NHS Foundation Trust, Southampton SO16 6YD, UK; 9 Leeds Institute for Data Analytics, School of Medicine, University of Leeds, Leeds LS2 9JT, UK; m.morris@leeds.ac.uk; 10 Department of Food Science and Nutrition, King Abdul-Aziz University, P.O. Box 42807, Jeddah 21551, Saudi Arabia; salbar1@kau.edu.sa

**Keywords:** 24-h recall, nutrition assessment, technology assisted dietary assessment, gestational diabetes

## Abstract

myfood24 is an online 24 hr dietary recall tool developed for nutritional epidemiological research. Its clinical application has been unexplored. This mixed methods study explores the feasibility and usability of myfood24 as a food record in a clinical population, women with gestational diabetes (GDM). Women were asked to complete five myfood24 food records, followed by a user questionnaire (including the System Usability Scale (SUS), a measure of usability), and were invited to participate in a semi-structured interview. Of the 199 participants, the mean age was 33 years, mean booking body mass index (BMI) 29.7 kg/m^2^, 36% primiparous, 57% White, 33% Asian. Of these, 121 (61%) completed myfood24 at least once and 73 (37%) completed the user questionnaire; 15 were interviewed. The SUS was found to be good (mean 70.9, 95% CI 67.1, 74.6). Interviews identified areas for improvement, including optimisation for mobile devices, and as a clinical management tool. This study demonstrates that myfood24 can be used as an online food record in a clinical population, and has the potential to support self-management in women with GDM. However, results should be interpreted cautiously given the responders’ demographic characteristics. Further research to explore the barriers and facilitators of uptake in people from ethnic minority and lower socioeconomic backgrounds is recommended.

## 1. Introduction

Gestational diabetes mellitus (GDM) has onset or detection in pregnancy, most commonly in the third trimester. Universal screening for GDM is conducted in some countries such as Australia. However, in the UK, screening targets those with one or more established risk factors. i.e., women with body mass index (weight kg/height m^2^) above 30, previous large baby >4.5 kg, previous GDM, ethnic family origin with a higher prevalence of diabetes, or a family history of diabetes [1]. It is performed at around 24–28 weeks gestation and uses an oral glucose tolerance test following fasting, to detect raised blood glucose levels.

The main treatment aim is to maintain healthy blood glucose levels throughout pregnancy to reduce the risks of complications for the mother and child; for example: macrosomia, stillbirth, and birth-related trauma [2]. After giving birth, blood glucose levels usually revert to normal; however, 50% of women with GDM will develop Type 2 diabetes within five years [1]. As with Type 2 diabetes, the treatment of GDM initially focuses on dietary and lifestyle changes with a healthy diet being recommended [1]. To achieve blood glucose targets, emphasis is placed on optimising the type of carbohydrate by encouraging low glycaemic index choices; and on reducing the portion size. If within one to two weeks of dietary modification, blood glucose levels are above the target range for pregnancy (fasting blood glucose below 5.3 mmol/L and one hour after meals below 7.8 mmol/L), women will then require medication for diabetes for the remainder of pregnancy; for example, metformin tablets or insulin injections. The self-management behaviours that are required to achieve these levels, including dietary modification, and frequent self-blood glucose monitoring, are demanding and are associated with increased levels of distress [3].

Standard care at diagnosis for women with GDM includes a dietary assessment, usually by a dietitian, from which education and negotiations for dietary changes will be based; progress may be reviewed at subsequent appointments with further dietary assessments [1]. As with other clinical populations requiring dietary management, typical dietary assessment methods include 24 h dietary recalls that are undertaken with a dietitian or with self-completion of paper food records. The level of detail acquired is variable, and it may include the frequency, amount, and type of food. Gathering and interpreting this information requires time and skills for both health professionals and patients. Such dietary assessment methods are known to have limitations. For example, interviewer-led 24 h dietary recalls can introduce reporting bias as subjects under-report certain foods due to social desirability [4].

The development and implementation of technology-assisted dietary assessment, including, for example the use of websites, mobile phone cameras, and mobile apps to log food intake, is proving to be an alternative to these traditional methods [5,6,7,8,9]. Such technologies in healthy populations are being shown to reduce costs [10], improve completion rates, and increase the accuracy of the dietary assessment [5,7,10,11,12]. Automated self-administered dietary assessment tool provides participants with more privacy than interviewer-led assessments; hence reducing judgment bias, which is associated with omissions in reporting and underestimation of portion sizes of unhealthy foods [13]. Therefore, minimising contact with the interviewer may encourage more participants to report all food items [14].

Given these favourable findings when using technology-assisted dietary assessment in healthy populations, their application in the management of medical conditions has begun to be explored [15,16]. This data can be instantly analysed and provide feedback to the user and health professional. These features can provide additional support and education outside of the clinic setting [15,17,18], and a study in primary care reported that technology-assisted dietary assessment may promote self-efficacy and increase empowerment in users [17]. Such tools also enable remote monitoring and follow up, potentially reducing the number of face-to-face appointments; therefore, there could be substantial clinical benefits and cost reductions associated with their use.

myfood24 is a self-completed online 24 h dietary recall tool, incorporating elements of an automated multiple pass methodology [19]. Foods consumed are entered on the system, by selecting from a list of options; portion size is then selected, and the food is added to a meal event or time. Prior to submitting the completed recall, it prompts the user to check for omissions, e.g., snacks. The recall is completed at one time, inputting all information from the previous 24 h period. The myfood24 food database contains 45,000 foods including both generic and branded items, 5600 with associated portion size images, and provides the user with an immediate nutritional analysis of their dietary intake. The tool was developed for large scale epidemiological studies in the UK population. Usability and acceptability testing of myfood24 has shown it is suitable to be used in healthy UK teenager (System Usability Score (SUS) Median score 80) and adult populations (SUS Median score 73) and relative validity testing in teenagers showed good agreement with interviewer-administered 24 h multiple-pass recall (MPR)s [19,20,21]. A validation study in healthy adults that includes the use of biomarkers has been undertaken [22]. Whilst myfood24 is currently used as a 24 h recall tool for nutritional assessment in epidemiological research, its application in healthcare as a food record has not been explored.

This study examined the introduction of myfood24 in a clinical population for the first time. Women with GDM, who are routinely required to complete food records, were requested to use myfood24 as an alternative tool for self-recording dietary intake. The aim of this observational study was to explore the feasibility of using the tool as an electronic food record and the usability of the tool software. Therefore, providing direction for the ongoing development and evaluation of this tool, both for use in clinical research, and ultimately, as an electronic food diary in routine clinical practice. The study also addressed wider aims, including the association between dietary components and blood glucose levels, and the broader experiences of women with GDM, which are the subject of other publications.

## 2. Materials and Methods

### 2.1. Ethics

All subjects gave their informed consent for inclusion before they participated in the study. The study was conducted in accordance with the Declaration of Helsinki, and the protocol and subsequent amendments were approved by the South Central Oxford Research Ethics Committee C (reference 14/SC/1267).

### 2.2. Eligibility

Women attending for their initial appointment following the diagnosis of GDM at Leeds Teaching Hospitals NHS Trust Diabetes in Pregnancy Clinic, were eligible to participate, provided they could read and understand English and were not to be commenced on any diabetes medication.

### 2.3. Recruitment

Women were invited to take part by a research midwife who provided a participant information sheet, addressed any questions relating to the study and secured informed consent to participate. The sample size of 200 was based on the sample size calculation for other research questions beyond the scope of this paper, examining the association between dietary components and blood glucose levels. This was a considerably larger sample than what was required for usability testing and examining the differences in usability based on sample characteristics [23].

A pragmatic approach was adopted for interviews, using a convenience sample of women who had experienced myfood24 in the main study. Guided by other qualitative research, a minimum of 12 interviews was anticipated as sufficient to provide saturation [24]. As the aim was to interview both participants who had used the tool and those who chosen not to, different recruitment methods were used. For those who had used myfood24, their interest could be indicated via the user questionnaire, and following a substantial amendment, those who had never completed myfood24, were sent an email. Informed consent for interviews was secured separately by the interviewer (ZD).

### 2.4. Study Design and Data Collection

**Figure 1:**
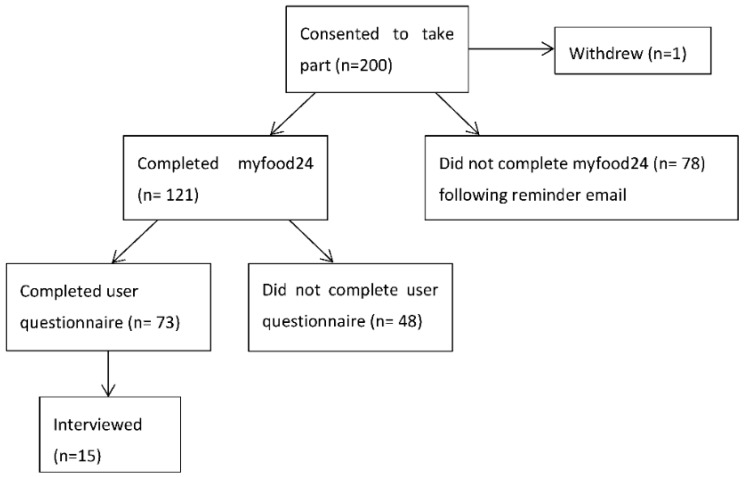
Study flow chart.

This was a mixed method prospective observational study. The stages of the study and the participation at each stage can be seen in Figure 1. Participants consented for relevant demographic and clinical data to be retrieved from their health records.

#### 2.4.1. Completing a Food Record Using myfood24

**Figure 2:**
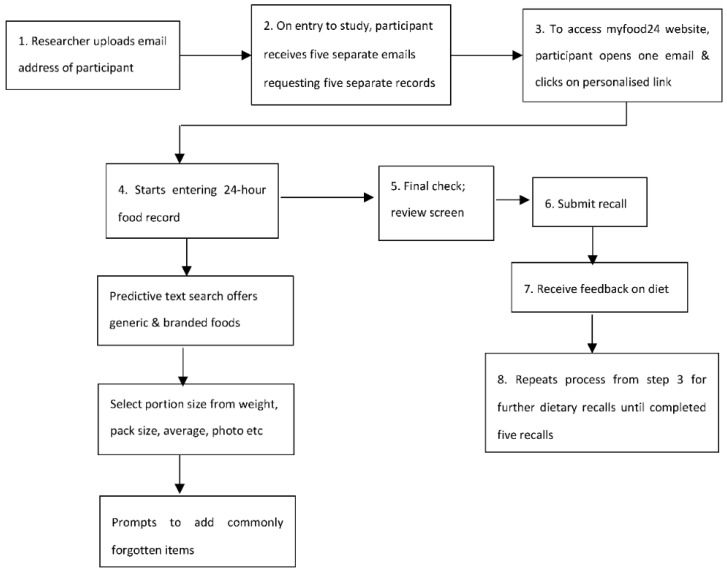
Process of accessing and completing an online myfood24 food record.

Antenatal care provision followed standard care guidelines for diabetes in pregnancy, which included ongoing self-monitoring throughout the pregnancy with blood glucose monitoring seven times a day and paper food diaries. By taking part in the study, participants were also requested to complete five 24 h food records in a two-week period using myfood24. The process of accessing and completing a myfood24 food record is illustrated in Figure 2. The request for five days of records was a pragmatic decision. This would allow women to record a large proportion of their habitual intake and to become more familiar with using myfood24; thus, providing more opportunities to test out the usability and functions of the website.

The feasibility of using the tool as a food record was investigated by measuring any online submission of a myfood24 food record and the total number of submissions per participant.

#### 2.4.2. User Questionnaire

Those who completed myfood24 on at least one occasion were emailed a link to complete the user questionnaire, administered by Bristol Online Survey [25]. The questionnaire consisted of multiple choice questions, Likert scales, yes/no responses and open-ended questions which covered demographic information, previous experience of using technology and food diaries, attitudes to technology and usability of myfood24. It included the SUS, a validated, reliable tool for measuring usability of the software [26]. This is a 10-item scale, with the users asked to rate their level of agreement with 10 usability statements (1 = strongly agree; 5 = strongly disagree) which gives a total score from 0–100. Scores below 50 are unsatisfactory with 50 to 70 are judged as marginal and above 70 considered good [27]. In practice, a SUS score above 68 is considered to be above average and anything below 68 is considered to be below average, and therefore the aim was for the SUS score to be at least 68 [28].

Usability considers user experience of the system, including the ease of understanding and ease of use of the software [29]. The SUS score from the user questionnaire, along with the interview data was the main measure used to assess usability of myfood24, when utilised as an online food record.

#### 2.4.3. Interviews

Interviews were semi-structured and followed a pre-designed flexible topic guide, exploring women’s views and experiences of using myfood24. The interviews sought to contextualise the completion rates and findings from the user questionnaire in the words of the women, exploring beyond the practical aspects relating to feasibility and usability. Women were interviewed at a time and location of their choice. All were interviewed by telephone with interviews lasting on average 35 min (range 20–54).

### 2.5. Data Analysis

#### 2.5.1. Statistical Analyses

Analyses were performed using Stata IC14 statistical software. Descriptive statistics were used to define the sample characteristics. Comparisons were made using chi-squared test for categorical data and independent *t*-test for continuous data. For all inferential statistics the significance level was two-sided and set at 0.05.

Further analysis, using data from the health records of the study population along with additional data from the user questionnaire, considered the secondary aim. This was to identify specific factors that may influence the usability of myfood24, such as demographics, other participant characteristics including online literacy, and features of the tool.

#### 2.5.2. Analysis of Interviews

Interviews were audio-recorded and transcribed verbatim in an anonymous format. Data were analysed using thematic analysis, using the principles that were outlined by Braun and Clarke [30], familiarising selfwith the data, generating initial codes, searching for potential themes, reviewing themes, defining and naming themes, and producing the report.

## 3. Results

### 3.1. Characteristics of Participants

Two hundred women consented to the study, and one of these withdrew. The mean age was 33.3 years (SD 5.0). According to health records, 76% of the women were overweight or obese at booking (first formal antenatal appointment), and the mean body mass index (BMI) was 29.7 kg/m^2^ (SD 6.5). Most women were White (57%) and one-third (33%) were Asian, and 36% women were primiparous.

### 3.2. Completion of myfood24 Recalls

**Figure 3:**
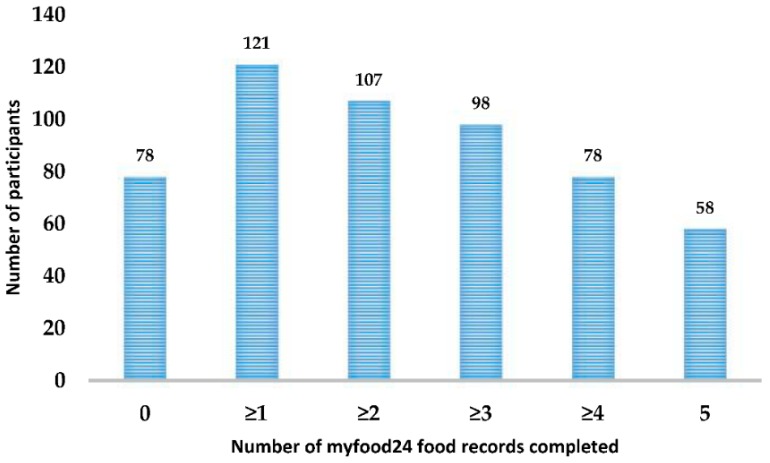
Number of myfood24 food records completed by participants (*n* = 199).

Women were asked to use myfood24 to complete and submit five online food records over a two-week period. For the full sample (*n* = 199), the mean number of days completed was 2.3 (SD 2.2) days; however only 61% (121/199) completed myfood24 at least once. In the 121 women who completed myfood24 at least once, the mean number days completed was 3.8 (SD 1.4); 98 (81%) completed it at least three times and 58 (48%) completed it all five times. Further details are shown in Figure 3.

There were statistically significant demographic differences between those who did (121/199; 61%) and did not (78/199; 39%) complete myfood24 at least once, as shown in Table 1. Women who used myfood24 as a food record were statistically more likely to have lower fasting blood glucose levels (*p* = 0.008), be of White ethnicity (*p* = 0.001) and primiparous (*p* = 0.02).

Within the women who completed myfood24, no statistically significant differences in demographic characteristics were found according to the number of times that it was completed, with comparison between women who completed it one to two times (*n* = 23), versus three times or more (*n* = 98).

### 3.3. User Questionnaire

User questionnaires were completed by 73 of the 121 women who used myfood24, a response rate of 60%. Comparisons were made between these women (*n* = 73) and those who did not return the questionnaire (*n* = 48), as shown in Table 2. Statistically significant differences in demographics were found; responders were older (*p* = 0.01) and had lower BMI at booking (*p* = 0.008). The mean number of myfood24 food records completed was significantly higher (*p* < 0.001) in those who completed the questionnaire.

The user questionnaire contained the usability questionnaire, which provided a SUS for measuring the usability of the software [26]. Therefore, this was only accessible to 121/199 participants, and of these, only 73 completed it; hence SUS was completed by 73/199 (37%) of the participants. The mean SUS score for myfood24 in this sub-group was considered to be good at 70.9 (95% CI 67.1, 74.6). Further questions on the user questionnaire provided the following demographic details on this sub-group. Of the 73 women, 47% were employed in managerial or professional occupations, and 58% were educated to degree level or above. Most women (90%) described themselves as being confident with using technology, with good access and the ability to use the internet (97%). Previous use of technology to record food intake was reported by 45%.

### 3.4. Interviews

#### 3.4.1. Characteristics of Interviewees

Despite a substantial amendment part-way through the study, to ensure that the invitation for interview was sent to those who did not complete any myfood24 food records, there were no expressions of interest to be interviewed in this group. Sixteen women who had completed myfood24 expressed an interest, resulting in 15 interviews with one declining due to time commitments. Characteristics of those interviewed were similar to the other participants who completed myfood24. The mean age was 35.1 years (SD 4.5); five were primiparous. Most women were White (13 White, two Asian). The mean BMI at booking was 28.3 (SD 4.7). All had completed myfood24 at least once, and 10 had completed it five times. The mean SUS score was 74.0 (SD 23.0).

Most women reported completing the record at the end of the day in the evenings, with a number of them using their clinic paper food records as an aid to recall that day’s food intake.

#### 3.4.2. Themes

Thematic analysis relating to usability and acceptability of using myfood24 as a food record identified the following themes: (1) ease of use, (2) impact on food choices, (3) comparisons with clinic paper records, and (4) future developments. Due to the characteristics of the interview sample, these themes represent only the views of women who had completed myfood24.

(i) Getting Acquainted: Ease of Use

Many of the women had used similar technologies before to record their diet and reported finding myfood24 to be comparable once they had become acquainted with the technology. Most found myfood24 ‘straightforward’ and that inputting data became faster with practice, initially taking approximately 20 min.

‘I found it quite easy… I mean, you just typed in a word and it would bring what you wanted up and then you chose from the list’.

(Interview 3)

Several features were reported that helped or hindered the ease of use of myfood24. Features of the tool that were most commonly identified as being useful for input were the food photographs to help with portion estimation, and the use of reminder prompts. Some also used the recipe function and favourites list; most reported that they would use these functions on a longer-term basis. The food database received mixed feedback. Some found the food database too restrictive, whereas others found the choice overwhelming. Some reported finding food entry to be burdensome when cooking from raw ingredients, and the process was too time consuming to be feasible.

‘Obviously you don’t weigh your food… it was nice to see actually a portion size’

(interview 6)

‘I didn’t have to go back and correct anything really because it reminded me’

(interview 3)

‘For something that was quite simple, it would take actually a long time to find it’

(interview 4)

‘It was quite time consuming having to kind of search for things and put everything in in its own, you know, like all the different components that made up a meal’.

(interview 11)

One reported disadvantage was the need to record all food entries for the 24 h period in one go, rather than being able to add data after each meal, making myfood24 less easy to use than paper diaries. Interviews identified that accessibility was hindered by problematic initial access to the myfood24 website, offering insight into the completion rates observed. As shown in Figure 1, myfood24 uses a web link accessed from the participant’s email account, with each recall requiring a separate email. Women reported that the process had been confusing and it was difficult to keep track of the multiple emails, all of which were sent on the day of entry to the study and without any indication as to which of the five days they referred to.

(ii) Feedback: Impact on Food Choices and Behaviours

On completion of a food record, myfood24 provides an instant summary of its nutritional composition, including energy, protein, fat, carbohydrate, fibre, salt, and micronutrients. This is compared to a dietary reference value.

All of the interviewees reported finding this feature useful to obtain feedback on their food intake and associated eating behaviours, although a minority questioned the accuracy. Women described how this feedback increased their knowledge of their dietary intake, how it could provide reassurance, and how it could enable women to consider what changes were required. Some women reported that the summaries were a motivating factor, influencing future choices about food types and portion sizes.

‘I did find it frustrating, to be honest, in terms of just trying to find what matched what I was eating, I don’t know how accurate it was for me’

(Interview 4)

‘a week’s worth of days in front of you it does make you think about what you’re eating and how much’

(Interview 3)

‘I was like ooh I shouldn’t have eaten that or oh, I’ve had a really good day today.’

(Interview 12)

Most women focused on the data relating to total carbohydrates and sugars—key to the management of GDM—but some also noted using the summaries to understand their fat and salt consumption. In addition, for some, the summary information highlighted that they had actually been too restrictive with their diet, in response to their diagnosis.

‘Once you can see it in numbers and can see the picture of it, it’s harder just to shrug off and think I’m fine...I couldn’t just go on with it’ (interview 11).

(iii) Comparing Online Self-Monitoring to Clinic Paper Diaries, and the Use of Real-Time Tracking

Comparing myfood24 with paper food diaries that women were required to complete as standard care, some felt myfood24 led to more accurate recording of food intake due to its use of reminders (for example prompting about snacks between meals, and the use of condiments) and recording of quantities, which had not been requested in as much detail in their paper food diaries.

‘I thought that [myfood24] made me remember things. It was more specific. I think it was easy to forget when you’re writing it down. You know because it reminded you—have you remembered to put a drink down here, have you remembered to put a snack down there.’

(Interview 9)

Women felt that whilst myfood24 was more accurate than paper-based diaries, in its current format it was also less convenient than paper-based diaries, particularly given the need to track blood glucose levels alongside the dietary intake in the paper-based diaries. Hence, although women valued the myfood24 summary information highly, the utility of myfood24 was limited by being produced at the end of a 24 h period and without any connection to blood glucose readings, limiting its use as a self-monitoring tool for improving blood glucose levels.

(iv) Future Developments: Suggestions based on Experience

Several suggestions were made by women, the most common being to improve accessibility via an app so that data could be conveniently logged throughout the day using a smart phone or tablet. Other suggestions for practical aspects relating to usability and improving its acceptability as an electronic food record were a larger food database, an option to scan bar codes, and easier retrieval of favourite foods. Women made several suggestions to adapt myfood24 to develop its accessibility, and also to develop its usefulness in promoting self-management and supporting behaviour change. Of greatest priority for this clinical population was the need to record blood glucose readings alongside dietary information, in order for the information to be meaningful and to develop knowledge of the impact of different types and quantities of food on blood glucose levels.

Some also identified the need for improved visual displays of the summary nutritional information, to support behaviour change. Suggestions included the ability to track information across time and individualised targets for nutritional intake, rather than reference values. Women also emphasised their perceived additional benefit from being able to use the tool in a diary format, rather than solely as a 24 h recall. This related both to aiding recall, but also to the way in which women used feedback to guide future food choices and behaviour.

‘I think a phone app would be a lot easier…you’d just keep updating it as the day went along… it’d make it more helpful like that’

(Interview 12)

‘I don’t know if there was an option where you could save that information, and maybe it was something that I missed, but I would have found that useful as an ongoing thing, if you could save it…’

(Interview 7)

‘I thought what might be quite useful would be more like a graph … that shows like you’re here and this is what you really should be getting, aiming for.’

(Interview 2)

## 4. Discussion

This study has demonstrated that it is feasible to use myfood24, a tool previously only used for nutritional epidemiology studies, as an electronic food record in a clinical population. Usability of the software was scored as good within a sub-group of the study population. However, the lower response rates throughout the study and the lack of sample representativeness mean that these results should be interpreted cautiously.

The study has generated a number of suggested areas for improvement which could increase the uptake of myfood24 as a dietary assessment tool in clinical populations. It is important that these are applied where possible, and their impact further evaluated. These changes, along with the ideas for improving the myfood24 software, should be considered prior to future studies examining its use as a dietary management tool to support behaviour change, in a healthcare setting.

### 4.1. Feasibility of Using myfood24 as An Electronic Food Record

The feasibility of using the tool as an electronic food record was investigated by measuring the total number of submissions of a completed myfood24 food record per participant.

In this study, 78/199 women (39%) did not submit any myfood24 food records. Reasons for this are not known and may extend beyond the acceptability of the tool as an electronic food record. It is noted that statistically significant differences existed between those who did and did not complete myfood24, some of which may be indicative of levels of motivation and self-management; unfortunately, further comparisons were precluded by certain data only being available for those who completed myfood24 and who went on to complete the user questionnaire.

Of those who completed the user questionnaire (*n* = 73), 45% of participants had used technology previously to log food, so this indicates it is common for women in this group to have similar previous experiences, potentially influencing acceptability in this is population. After participants used myfood24 at least once, the majority went on to use it again, with almost half of women completing it the five times as requested; which does suggest that it is feasible to use this tool as an electronic food record. There did not appear to be any demographic differences regarding number of times completed, however a larger sample size may be needed to determine this. Findings from the interviews suggested that there was confusion experienced with the email system. This system had remained unchanged from its application in nutritional epidemiology data collection and appeared not to suit this setting. As a consequence, this could have impacted on the uptake. In contrast, the data suggests that its perceived provision of a more complete and accurate record and the receipt of the nutritional intake summaries immediately following the diary submission, made a valuable contribution to its acceptability, and enhanced its use as a food record. This is supported in other studies [15,18]. Future work should prioritise these areas for development.

Women continued to have to complete paper-based food and blood glucose diaries as part of standard care. Some women found the paper diary more convenient, due to the ease of access and having all the data in one place, enabling links to be made between food intake and blood glucose levels. Studies in populations with diabetes and renal disease support the suggestion that being able to input foods in real time via a phone app and adding other clinical data such as blood glucose levels can improve acceptability [15,16]. The absence of this functionality may have affected completion rates, as may have the requirement to dual record data on both paper and electronic records.

myfood24 user data can be shared with both the user and the provider/health professional; hence it has the potential to promote user–provider interactions. This function was not utilised in this study. Significant benefits in diabetes outcomes have been found when patients were provided with analysis or feedback from clinicians on their data [15]. Additionally, acceptability and effectiveness of a smartphone nutritional assessment tool for healthy pregnant women, was enhanced by using a similar feature [18]. Therefore, utilisation of this function should be a priority in future studies and its impact on the uptake of the tool assessed.

### 4.2. Usability

Usability considers the user experience of the system, including its ease of understanding and ease of use [29]. This was measured using the SUS and interview data. The SUS score was classed as good (SUS = 70), and it was comparable to the scores obtained in the general population in other myfood24 evaluations with adults (SUS = 80) and adolescents (SUS = 74) [19,20]. However, it was only obtained in 61% of participants who used the system, and it is noted that this sub-group lacked representativeness compared to the whole study population. This lack of representativeness was also present in the group of interviewees, from whom further data on usability of myfood24 software was collected. Therefore, although this study has generated valuable insights into the usability of the software, that can be further explored and considered for future development of myfood24, no conclusions on the usability of myfood24 for this clinical population can be made at present.

### 4.3. Improving Uptake of Use of the Tool and Usability of Software in the Future

A number of suggestions were made by interviewees regarding how to improve myfood24 software. Many of these are applicable to all dietary assessment tools/ food logging applications [6,17] and include: improvements to speed up food data entry, extensions to the food database, including ethnic foods, chain restaurant meals and popular homemade recipes, and more support to estimate portion sizes. Specifically related to myfood24, women wished to input food in real time and not have to enter the entire day’s food intake in one entry. They requested being able to undertake food records as frequently as they wanted, to access previous records, to be able to utilise food lists and to also make comparisons between days. Recent myfood24 developments on the visual displays of nutrient summary feedback, could be adapted for the healthcare setting to address some of these suggestions. Further thought into how the website is accessed and the setup of personal accounts for clinical rather than research purposes, is crucial to achieving this, and improving uptake. This would reduce the confusion experienced with the current email system and allow women to undertake dietary recalls as frequently as they choose to; furthermore, personal accounts could be used to promote self-monitoring during the pregnancy and beyond.

The findings highlight the importance of tailoring tools to the needs of population, illustrated here by the importance of real-time tracking for women with GDM and need for integration of dietary intake with other data; here for blood glucose, but other conditions would benefit from recording other health information (e.g., weight, other blood test results, blood pressure, activity data). Continued development by the myfood24 consortium and website developers, taking into account suggestions for further improvements from this and other studies [6] will enhance the usability further.

### 4.4. Opportunities for Use of myfood24 in a Clinical Setting

This study has established that it is feasible to use myfood24 as an electronic food record in the GDM population, and provides helpful insights into how to optimise its use in this population. There could be significant benefits of introducing myfood24 in this clinical setting. Diabetes ante-natal care includes frequent follow up at lengthy multi-professional clinic appointments and by telephone. Therefore, health apps have been developed specifically for GDM to enable health professionals to manage case-loads using remote monitoring of blood glucose levels [31]. Online food diaries could be used to share data in the same way, and these could be beneficial for a number of reasons. In diabetes care, such tools have been shown to be a more engaging and accurate way of capturing a dietary assessment [5,16] and are preferred to traditional methods [32]. Women in our study commented that they began to consider changes in their food intake from performing the recalls and receiving the feedback; this has been observed in other populations [15,17], and has been shown to improve the uptake of self-monitoring and lead to dietary changes [33]. Following initial education, women could continue to monitor their dietary intake and receive feedback from the website which could result in increased self-efficacy and self-management skills. Additionally, as demonstrated in other studies, remote monitoring and provision of feedback by the health care professional could reduce workload during clinics and enhance service provision by freeing up time for education and support [12,34]. In other chronic conditions, use of dietary assessment technologies in this way has had a beneficial effect on clinical outcomes [16,35].

The interviews highlighted the emotional aspects of living with GDM, and the breadth of behaviours that women with GDM are tasked with managing (reported in full elsewhere: [36], in preparation) indicating that if myfood24 were to be optimised for mobile devices, there would likely be other beneficial components that may support women with GDM. From diagnosis of GDM, women are expected to begin to make immediate changes to their lifestyle to meet the blood glucose target recommendations. However, there will be a wide range of levels of knowledge, skills and confidence around healthy eating. From women’s feedback provided in interview, it appears that myfood24 has the potential to improve the health literacy and eating behaviours of women with GDM. This is something that would require further testing. Reducing dependence on the health care professional to advise on dietary changes, and increasing knowledge and skills, has been shown to increase an individual’s sense of control. Receiving prompts as cues for action and receiving feedback on their nutritional intake alongside biofeedback through blood glucose levels are recognised behaviour change techniques that can support healthy behaviours [37]. This increased level of control over their condition and perceived treatment efficacy may additionally help to reduce the levels of distress experienced [38,39].

### 4.5. Strengths and Limitations of the Study

There are several strengths to this study. There was a large study population, which provided a complete data set on 73 women. The qualitative element of the study provided valuable insight into the trial of the tool in everyday life, adding depth that has been reported as lacking in other studies of this type [6].

Limitations arose from the data collection methods in the study. The user questionnaire provided valuable information on the demographics and technological skills of the women, but it was only collected from 37% of the study population. In hindsight, if this had been completed at baseline, without the SUS, this would have provided significantly more information about the women who did not respond at the different stages of the study. Furthermore, there was a lack of evaluation built into the study to routinely establish reasons for their not using myfood24 even once. This could in part reflect the methods for accessing myfood24 (i.e., through automated emails), or challenges with usability. However, other reasons should have been considered such as how the tool was introduced to women. Increasing individuals’ understanding of the benefits of using myfood24, such as those highlighted in this study around accuracy and the receiving of feedback, could be important for increasing uptake, and should be built into future interventions. Additionally, it was not established how much of an impact being in the late stages of a clinical complex pregnancy affected uptake.

Another significant weakness was the lack of representativeness of the data. This included a lack of ethnic diversity, which diminished with each stage of the research. A smaller proportion of women from black and minority ethnic (BME) groups completed the user questionnaire and took part in the interviews, limiting transferability of the study findings. As GDM is more prevalent in women from BME groups, this is not representative of a typical GDM population in the UK [40], and the barriers and facilitators for using myfood24 in BME groups needs to be explored in future studies. Over-represented in the group who completed the user questionnaire were women who were well educated and who worked in professional jobs. Based on those completing the questionnaire, the study population appeared to be confident in using technology and had good access to the internet, as well as previous experience with similar technologies. Again, this limits the conclusions that can be drawn from the results.

Finally, once women had begun to use myfood24 they may have been willing to continue to use it throughout the pregnancy; however the system did not allow them to continue to access myfood24 beyond their five records. The opportunity for continued use would have provided valuable further insights into its use by participants with a health condition [16].

This study has generated a number of suggestions for the future development of myfood24 for clinical populations. Real-time data entry assisted by a phone app format, could improve acceptability and usability. Adding further features such as improving the presentation and content of the feedback, and tracking pertinent biomedical data alongside nutritional information, would increase its potential beyond dietary assessment, and are ready for testing in the context of self-management of health conditions. The feasibility of its application within the health service should be explored in a feasibility study that also captures the views of users and health professionals on its use as an alternative tool in dietetic care. Ultimately a definitive trial considering the clinical and cost-effectiveness of a refined myfood24 tool in clinical practice is recommended.

## 5. Conclusions

This study has demonstrated that it is feasible to use myfood24, an online 24 h dietary recall tool developed for nutritional epidemiology studies, as an electronic food record in women with GDM. Usability of the software was assessed as good by a subgroup of the women. However, a higher response rate could have potentially been possible with a more diversely representative sample; therefore, the barriers against achieving this should be explored in future research.

The findings have generated a number of suggestions to improve acceptance and to enhance the usability of such tools in clinical populations. Women were motivated to use the tool, largely due to the feedback on their nutritional intake it provided, although being able to record food intake and input clinical data (blood glucose levels) in real time using mobile-optimised technology was deemed necessary to be able to replace paper-based diaries. Issues arose from employing the tool in a different context to the one in which it had been designed for, causing practical problems which need to be addressed.

Following improvements to the tool, suggested in this study and from other research findings, myfood24 should then be further evaluated within a healthcare setting, exploring its use as a dietary management tool to enhance behaviour change.

## Figures and Tables

**Table 1:** Comparison of characteristics between women who completed myfood24 at least once (*n* = 121) and those who did not (*n* = 78).

	Completed myfood24 (*n* = 121)		Not Completed myfood24 (*n* = 78)		*p* Value
	*n*	Mean	*n*	Mean	
Maternal Age, years (SD)	121	33.5 (4.6)	78	33.2 (5.6)	0.7
Booking body mass index (BMI), kg/m² (SD)	119	29.5 (6.2)	77	30.0 (7.0)	0.7
Fasting Blood Glucose, mmol/l (SD)	115	4.9 (0.6)	61	5.2 (0.8)	0.008
**Ethnicity**					0.001
White (%)	79	65.3	35	44.9	
Asian (%)	31	25.6	23	29.5	
* Other (%)	11	9.1	20	25.6	
**Parity**					0.02
Primiparous (%)	52	43	21	26.9	
Multiparous (%)	69	57	57	73.1	

* Other ethnicities included Black, Chinese and Mixed race.

**Table 2:** Characteristics of myfood24 users who completed the user questionnaire (*n* = 73) and those who did not (*n* = 48).

	Completed User Q		Did Not Return User Q		*p* Value
	*n*	Mean	*n*	Mean	
Age, Years (SD)	73	34.3 (4.3)	48	32.2 (4.7)	0.01
Pre-Pregnancy BMI, kg/m^2^ (SD)	72	28.3 (5.0)	47	31.4 (7.5)	0.008
Fasting Blood Glucose, mmol/L (SD)	72	4.9 (0.7)	43	5.0 (0.5)	0.5
**Ethnicity**					
White (%)	52	71.2	27	56.3	0.4
Asian (%)	15	20.6	16	33.3	
Other (%)	6	8.2	5	10.4	
**Parity**					0.9
Primiparous (%)	31	42.5	21	43.8	
Multiparous (%)	42	57.5	27	56.2	
**Number of days completed myfood24 (SD)**	73	4.2 (1.1)	48	3.2 (1.6)	<0.001
**System Usability Scale (SUS) score (95% CI)**	73	70.9 (67.1, 74.6)	-	-	-
**Occupation**	68		-	-	-
Managerial & professionals (%)	32	47			
Intermediate & lower supervisory	17	25			
Semi routine & routine (%)	4	6			
Not employed (%)	15	22			
**Education level**	70		-	-	-
Degree or above (%)	42	58.3			
No degree (%)	30	41.7			
Had previously filled in food diary (%)	26	35.6	-	-	-
Previously used technology to record food (%)	33	45.2	-	-	-
Internet ability good to excellent (%)	71	97.3	-	-	-
Access to internet (%)	72	98.6	-	-	-
Use internet daily (%)	69	94.5	-	-	-
Confident in using technology (%)	66	90.4	-	-	-
